# Nanotubes of a Different Kind: Stoichiometry and Geometry of the Orange II/γ-Cyclodextrin Complex in Water

**DOI:** 10.1021/acs.chemmater.3c02518

**Published:** 2024-05-14

**Authors:** Mohan Srinivasarao, David W. Jenkins

**Affiliations:** † School of Materials Science and Engineering, Georgia Institute of Technology, Atlanta, Georgia 30332, United States; ‡ School of Chemistry and Biochemistry, Georgia Institute of Technology, Atlanta, Georgia 30332, United States; § FHI 360, Durham, North Carolina 27713, United States

## Abstract

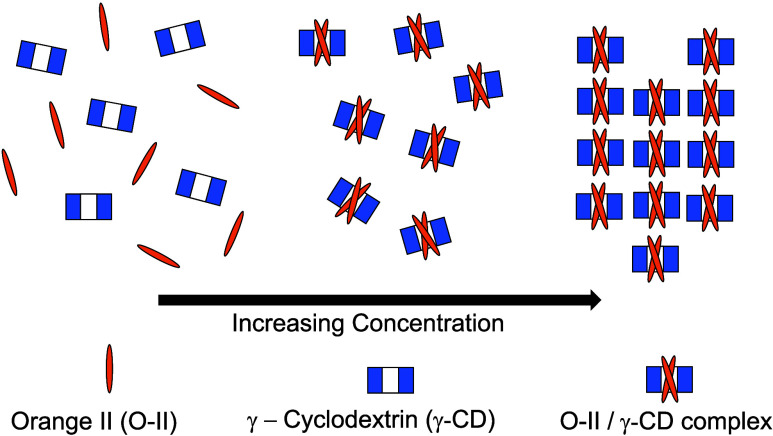

Orange II (O-II), a water-soluble ionic azo dye, aggregates and eventually forms needle-like crystals at concentrations greater than 0.15 M. However, when equimolar amounts of γ-cyclodextrin (γ-CD) are added to solutions containing O-II at 0.025 M or higher, the solution’s appearance rapidly changes presenting a viscous, birefringent liquid, a lyotropic liquid crystalline solution. Birefringence is absent when viewing aqueous solutions of only O-II or γ-CD at concentrations greater than 0.03 M. Using ultraviolet–visible (UV–vis) and fluorescence spectroscopy, coupled with conductivity measurements, we postulate a structure for the basic “building block” of the self-assembly that eventually gives rise to a rodlike superstructure, leading to the formation of a lyotropic liquid crystalline phase.

## Introduction

Inclusion complex formation of host cyclodextrins^1,2^ with guest hydrophobic molecules in water is an area of research important in the study of supramolecular chemistry,^3^ the development of pharmaceuticals, cosmetics, and pesticides, among others.^4^ Cyclodextrins (CDs) are cyclic carbohydrates comprised of 1,4-α linked glucose repeat units generated from the enzymatic degradation of starch. These molecules possess a toroidal shape with a hydrophilic exterior surface and a hollow hydrophobic cavity, and are able to host “guest” molecules with a defined size, shape, and stoichiometry of interactions. The most common types are α, β, and γ which contain 6, 7, and 8 glucose units, respectively; however, larger cycles do exist.^5−8^ In addition to the high water solubility of CDs, the CD inner cavity is considered to be hydrophobic, promoting the inclusion of molecules with low water solubility.

**Figure 1:**
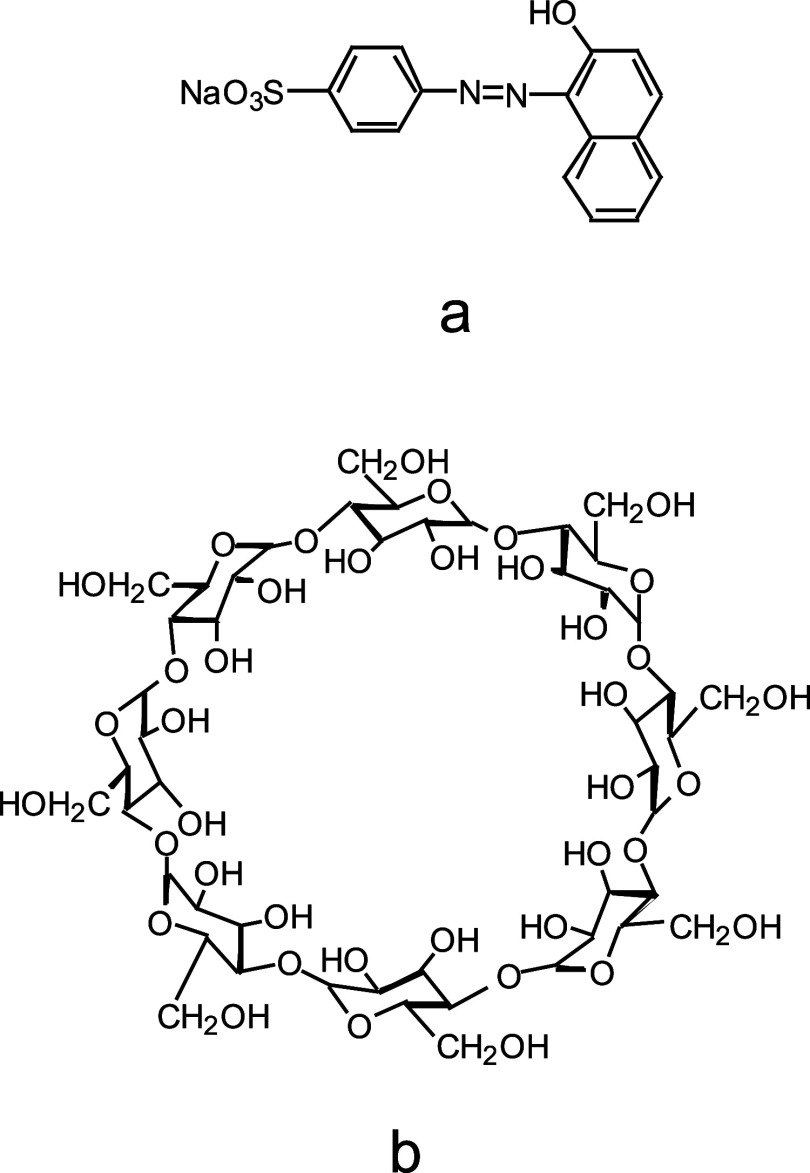
Chemical structures of O-II (a) and γ-CD (b).

In regard to the supramolecular chemistry of CDs, Suzuki and Sasaki reported that a lyotropic liquid crystalline phase forms when Orange II (Figure 1a) and γ-cyclodextrin (Figure 1b) are dissolved in water.^9,10^ For equimolar solutions, an anisotropic phase is readily observable when the concentrations of each compound are greater than ∼0.025 M, and more importantly, the absence of either of the components (CD or O-II) does not result in an anisotropic phase. In order for a liquid crystalline phase to appear in solution, one requires a solute with large enough geometrical asymmetry, common examples being rodlike polymers in solution. Thus, it is obvious, then, that the two different components (CD and O-II) must self-assemble with one another to build a larger species with the required geometrical asymmetry in solution. It is possible that this system is very similar to lyotropic liquid crystalline systems which are observed with surfactants,^11,12^ or lyotropic chromonic liquid crystals (LCLCs)^12^ in the sense that a “building block” is utilized in solution to construct a larger species with geometrical asymmetry. Understanding the complexation of O-II with γ-CD in a relatively dilute solution (meaning to determine the existence and the nature of a building block for this system) is vital to deciphering the mechanism of the formation of the anisotropic phase. To this end, Suzuki et al.^10^ proposed that a rodlike structure forms where an elongated stack of γ-cyclodextrin molecules form a single cylindrical channel capable of hosting Orange II in some manner down its length. Clarke et al. studied the complexation of O-II with γ-CD using ultraviolet–visible (UV–vis) spectroscopy and suggested that an O-II dimer is included within the cavity of γ-CD,^13^ thus providing a basic building block for the geometrically asymmetric structure.

Primarily, the purpose of this work is to provide additional evidence from fluorescence and conductivity measurements, which support the inclusion of an O-II dimer within the γ-CD cavity. Second, inclusion complex geometries are proposed based on induced circular dichroism spectra of O-II (found in the literature) and molecular exciton theory, which provide further detail as to the O-II orientation within the γ-CD cavity. Finally yet importantly, we propose a superstructure that is rodlike which will lead to the observation of a lyotropic liquid crystalline phase above a critical concentration.

## Experimental Section

### Materials

O-II (10 g, Aldrich 87%, *M*_W_ = 350.3 g/mol) was dissolved in 50 mL of distilled water at 85 °C. The dye was precipitated upon addition to 300 mL of ethanol, filtered, washed with 150 mL of ethanol, and dried. ^1^H NMR (Varian Gemini 300 MHz spectrometer) of the purified O-II indicated the only protons present where those assignable to the dye. γ-Cyclodextrin (Wacker-Chemie, Cavamax W8 Food grade, 98% dry weight, *M*_W_ = 1296 g/mol) was used as received. The purified O-II and γ-CD were sent to Atlantic Microlabs (Norcross, GA 30091) for elemental analysis (EA): O-II: Theoretical C – 54.8%, H – 3.14%, and N – 7.99%; Found C – 51.5%, H – 3.6%, and N – 7.55%; γ-CD: Theoretical C – 44.4% and H – 6.2%; Found C – 40.5% and H – 6.3%. The discrepancy between the observed and theoretical EA is attributed to the presence of moisture in both samples. From the EA data, the purities for O-II and γ-CD were determined to be 94.6 and 91.3%, respectively.

### Methods

Optical micrographs were obtained using a Leica DMRX polarized light microscope with a Sony DKC-5000 Digital Photo Camera. Sample cells were constructed by spacing the coverslip and the microscope slide with a 200 μm layer of a Teflon tape. Cells were sealed with water-resistant epoxy. Fluorescence spectra were obtained from a steady-state Photon Technology International fluorescence spectrophotometer, which uses a Model 814 PMT photon-counting detector. Slits were adjusted to a spectral bandwidth of 5 nm, and the spectra were measured at ambient temperature with a 3 s integration time and a 2 nm step size. Spectra at 5 × 10^–5^ M O-II were measured in a quartz cell with an incident path length of 0.2 cm and an emission path length of 1 cm. Spectra at 0.05 M O-II were measured in sample cells constructed from glass plates (12.5 mm × 75 mm) which were sandwiched together using a 300 μm Teflon spacer and sealed with water-resistant epoxy. Cells were placed in the cuvette holder at a NE–SW orientation to the excitation beam. Conductivity measurements were made at 25 °C in deionized water using an Accumet Research AR50 unit with an Accumet Conductivity Cell (1.0 cm^–1^ cell constant) standardized with a Fisher 1000 μS/cm calibration solution.

## Results

**Figure 2:**
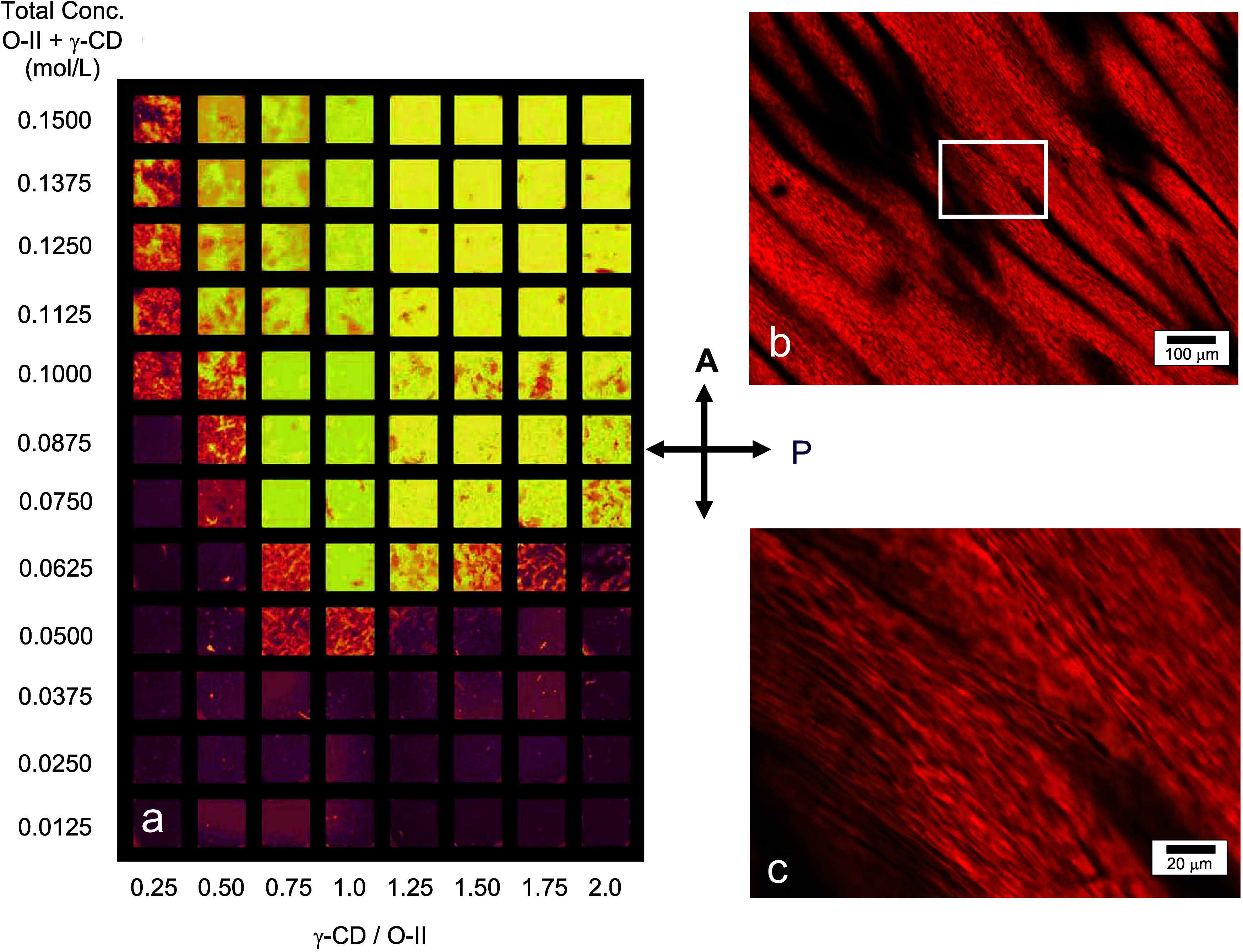
Unmagnified image (a) of various solutions in a 96-well plate at different ratios and concentrations of O-II and γ-CD that were illuminated between crossed polarizers. Optical micrographs (b, c) of a sample of 0.05 M (aq.) O-II and 0.05 M (aq.) γ-CD taken between crossed polarizers at different magnifications. Sample position is identical in both micrographs. Image c is a magnification of image b from within the white box.

From Figure 2a, the highly birefringent nature of the aqueous mixture of O-II and γ-CD is shown more generally for a range of concentrations and stoichiometric ratios, where the equilibrium-driven nature of complexation occurs more readily with equimolar ratios at and above a total concentration of 0.05 M. Choosing a 0.05 M O-II and 0.05 M γ-CD solution (well within the region where high levels of birefringence can be observed), optical micrographs (Figure 2b,c) show the striated nature of the anisotropic regions. With 0.05 M O-II, no birefringence was observed with the presence of 0.05 M α-CD or 0.05 M β-CD in the same manner as with γ-CD (although a few traces of small birefringent spots were slightly noticeable with β-CD at the same magnification as seen in Figure 2b).

**Figure 3:**
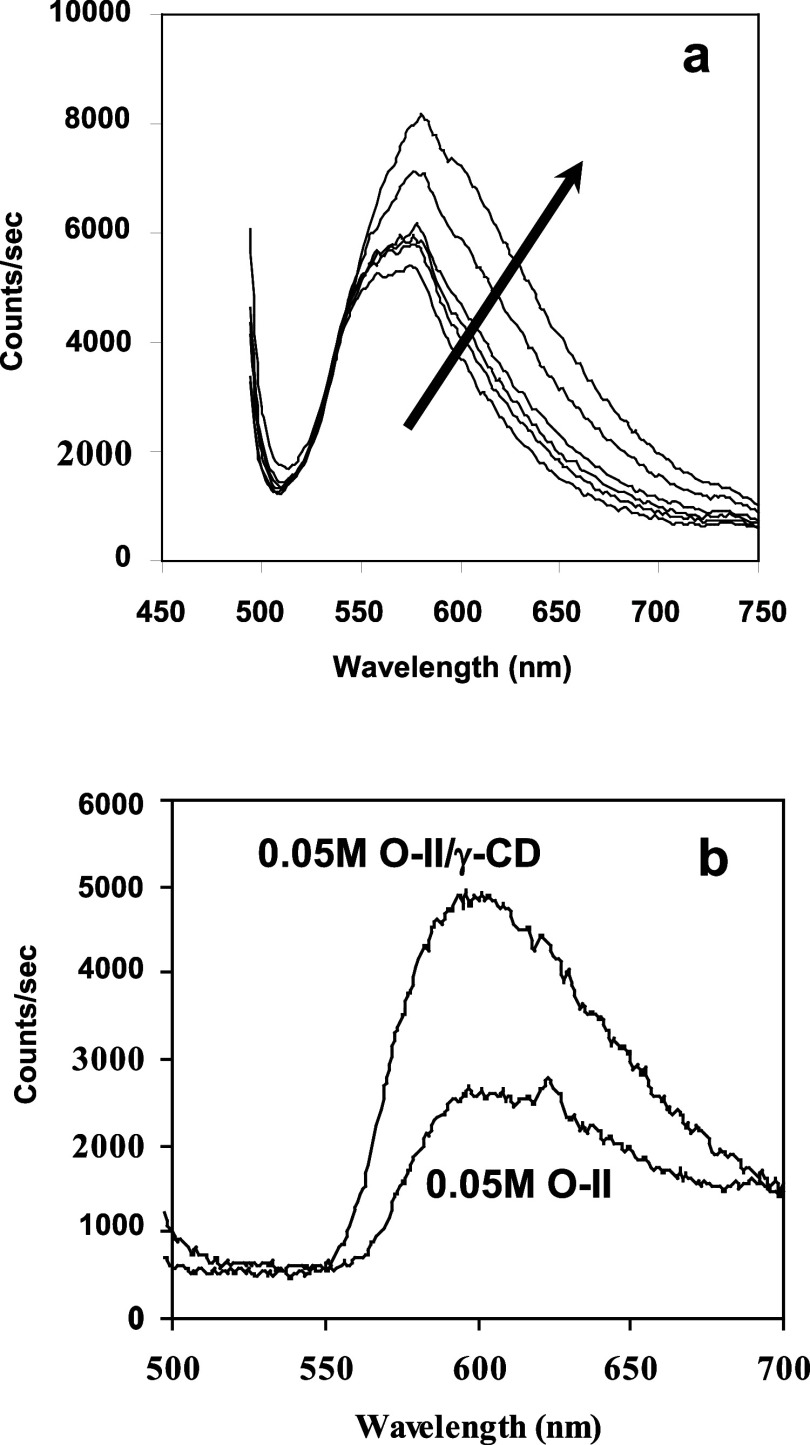
(a) Fluorescence spectra for Orange II at 5 × 10^–5^ M (aq.) with increasing amounts of γ-CD with 484 nm excitation. The arrow indicates an increase in the mole ratio of γ-CD/O-II in the following manner: 0, 0.05, 0.10, 0.20, 0.50, 1.0. (b) Fluorescence spectra for 0.05 M (aq.) O-II and 0.05 M (aq.) O-II/γ-CD with 484 nm excitation.

Figure 3a presents the fluorescence spectra observed for O-II (5 × 10^–5^ M) in the presence of increasing amounts of γ-CD with 484 nm excitation (484 nm is the λ_max_ for O-II in the visible spectrum). Determining the true emission maximum for these spectra is somewhat distorted due to the presence of the Raman line for water at ∼580 nm. Ibanez et al. report the emission maximum for O-II to be 560 nm,^14^ which is in reasonable agreement with the emission spectrum for O-II (without γ-CD) shown in Figure 3a. The main effect of the addition of γ-CD is the emission red-shifts to approximately 590–600 nm and increases in intensity. As depicted in Figure 3b, increasing the dye concentration to 0.05 M results in an emission maximum in the range of 590–600 nm either with or without γ-CD present, where the emission is more intense with γ-CD present.

**Figure 4:**
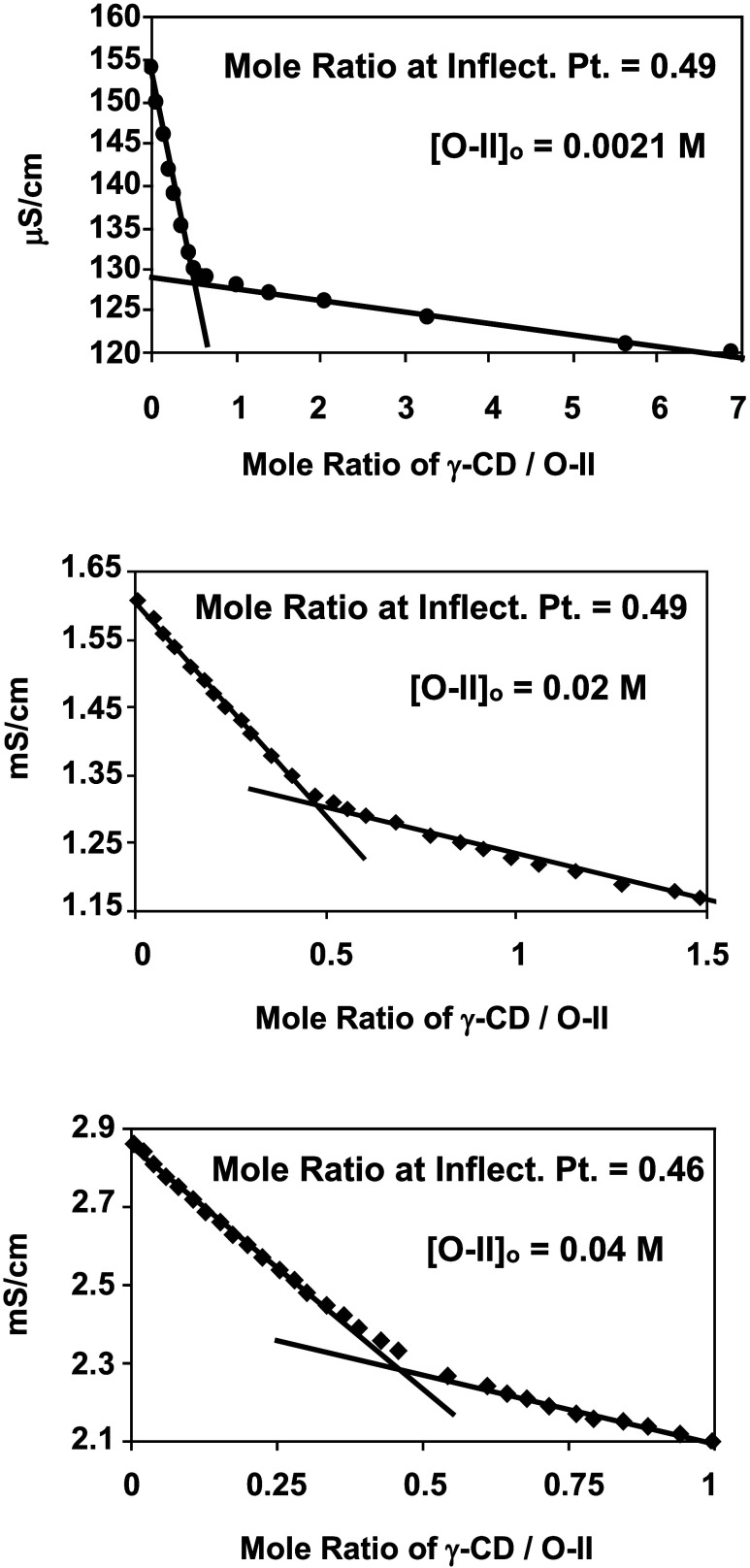
Conductometric titrations of O-II (aq.) with powdered γ-CD as the titrant.

Figure 4 shows the results for conductometric titrations of aqueous O-II solutions, at initial dye concentrations of 0.0021, 0.02, and 0.04 M, where powdered γ-CD was used as the titrant. In each case, the curves indicate a relatively strong decrease in solution conductivity with the addition of γ-CD, followed by a weaker decrease in conductivity after an inflection point that corresponds to a mole ratio of γ-CD to O-II of ∼0.5. The sharpness of the inflection is quantified by the ratio of the slope of the initial region to that of the latter. The slope ratios for the titrations having initial dye concentrations of 0.0021, 0.02, and 0.04 M are 35.2, 4.5, and 3.6, respectively.

## Discussion

### Fluorescence and Conductivity Measurements

**Figure 5:**
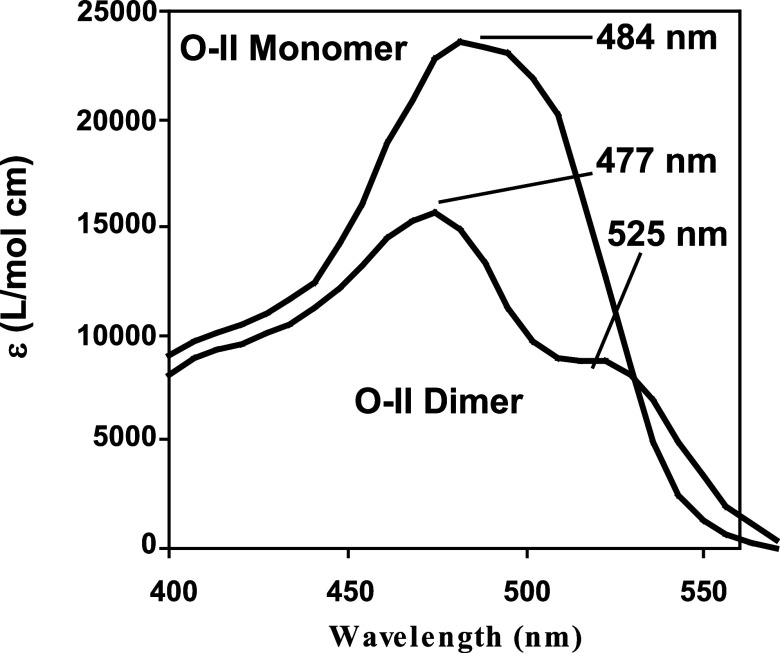
Monomer and dimer spectra for O-II as calculated by Reeves et al.^20^.

Owing to its amphiphilic nature, O-II is known to aggregate in water with increases in concentration, where aggregation occurs more strongly with increases in ionic strength.^15−18^ Dye aggregation is generally considered to proceed through the planar stacking of the dye in solution.^19^ Small-angle X-ray scattering studies of 0.02 M O-II (aq.) measured a solute *M*_w_ of 602,^18^ indicating the formation mainly of O-II dimers in equilibrium with O-II monomers (*M*_w_ = 350) and possibly higher-order aggregates. Reeves et al. studied the monomer/dimer equilibrium of O-II in water with UV–vis spectroscopy as a function of dye concentration.^20^ The major spectral changes observed with increases in concentration are that the molar absorptivity of the 484 nm band decreases and begins to blue-shift, with an isosbestic point at 530 nm. The factor analysis method utilized in this study led them to suspect that higher-order aggregates of O-II existed above 1.0 mM. Within the concentration range of 0.004–1.0 mM, absorption curves of pure monomers and pure dimers in extinction units were calculated, which allowed a reconstruction of the experimental absorption curves due to contributions from monomer and dimer O-II. Figure 5 shows the monomer and dimer spectra for O-II determined by Reeves et al.,^20^ which will be utilized in later discussions of alternative dimer geometries.

In their UV–vis study of the complexation of O-II with γ-CD, Clarke et al.^13^ observed that for a constant concentration of O-II, the addition of γ-CD resulted in the same visible spectral changes as Reeves et al.^20^ observed when the concentration of O-II was increased in the absence of γ-CD. Clarke et al. concluded that the major type of complex formed is that of an O-II dimer included within the γ-CD cavity.

From Figure 3, a 30–40 nm red shift in O-II emission observed when the dye concentration increased from 5 × 10^–5^ M to 0.05 M in the absence of γ-CD is consistent with an excimer emission from dye aggregation due to planar stacking with increasing dye concentration. At a more dilute dye concentration, the addition of γ-CD causes the red shift in the emission, suggesting its promotion of dye aggregation. Increases in the emission intensity observed with the addition of γ-CD for either dye concentration could be due to an environmental shielding of the dimer due to inclusion, reducing the amount of external conversion with the solvent. However, Conners points out the somewhat ambiguous nature of interpreting changes in fluorescence spectra with the addition of CDs because issues of molecular rigidity, environment polarity, and fluorophore collisions are all important.^21^

Conductometric titrations have been used to study the complexation of ionic organic molecules with cyclodextrins.^22−27^ The premise behind this method is that the conductivity of a solution containing a constant amount of ionic guest will decrease with the addition of cyclodextrin. This is due to the fact that the inclusion of the dye causes a decrease in the diffusivity of the conductive species.

The position of any inflection point observed in the titration curve has been used to interpret the stoichiometry of the complex,^25−27^ while the sharpness of the inflection point is related to the calculated strength of the binding constant; this point is well demonstrated in the work by Junquera et al.^27^ Although inclusion is considered to be the primary source for decreases in solution conductivity, the addition of cyclodextrin to an aqueous solution of inorganic electrolyte can cause a reduction in conductivity even though the electrolyte is not believed to bind or be included within the cavity. This more secondary effect, attributed to a viscosity increase upon the addition of cyclodextrin, was acknowledged in binding studies of ionic biphenyl compounds with α-CD,^22^ where the decrease in conductivity of aqueous NaCl solutions when titrated with α-CD was quantified and used as a correction for determining the binding constant.

Regardless of initial dye concentration, the titration curves presented in Figure 4 each indicate an O-II to γ-CD stoichiometry of 2 to 1 as a result of the inflection point at a mole ratio of γ-CD/O-II of ∼0.5. The sharpness of the inflection points (indicated by the slope ratios) is observed to decrease with increases in dye concentration. Based on the work by Junquera et al.,^27^ this would suggest that the calculated binding constant for this system would be expected to decrease as the dye concentration increases. Although these types of calculations will be the subject of future research, an explanation for this observation could be related to the increase in ionic strength, which occurs with increasing dye concentration. As alluded to previously, this might promote the formation of higher-order aggregates and subsequently diminish the driving force for inclusion within the γ-CD cavity. Nonetheless, it must be noted that the inclusion of an O-II dimer within the γ-CD cavity is the most likely complex formed in this system and can be considered the “building block” for the superstructure that eventually leads to the formation of a lyotropic liquid crystalline phase.

### Geometry of the O-II/γ-CD Complex

**Figure 6:**
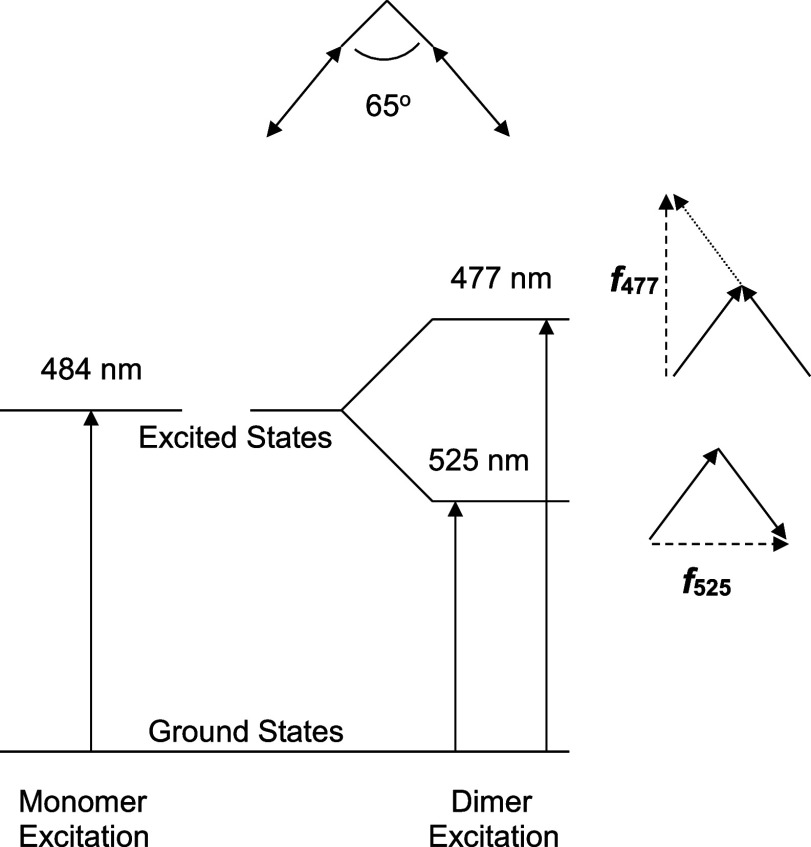
Energy diagram for oblique transition dipoles at an angle of 65° according to exciton theory^28^ for the O-II dimer.

In order to consider dimer geometries and their orientation within the γ-CD cavity, an interpretation of the O-II monomer and dimer spectra shown in Figure 5 is warranted. Using the λ_max_ for each band in the spectra, the energy-level diagram for the O-II dimer in Figure 6 can be generated according to the molecular exciton theory for oblique transition dipoles.^28^ In regard to dimer excitation, the splitting arises from the bidirectionality of the transition moment that induces a higher-energy transition (like charges in the same vicinity) and a lower-energy transition (unlike charges in the same vicinity). The intensity of absorption or the oscillator strength (*f*) is related to the vector addition of the dipoles for the given transition. Note that the orientations of the new transitions are perpendicular to one another. Reeves et al. deconvoluted the monomer and dimer spectra which allowed the calculation of oscillator strength *f*, for a given absorption based on eq 1.^20^
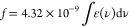
1From the calculated values of *f*_525_ and *f*_477_, the angle Θ between the two monomer transition dipoles was determined to be 65° by Reeves et al.^20^ (used as the oblique angle in Figure 6) with eq 2.^29^
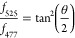
2Reeves et al.^20^ stress the possible inaccuracy of the angle calculation due to complications that occurred during deconvolution from the azo/hydrazone tautomer equilibrium^30^ which exists for O-II. Nonetheless, this angle value will be used as a guide for considering dimer geometries and their orientation within the γ-CD cavity.

Induced circular dichroism of included achiral guests within CD cavities can be used as a means to evaluate guest orientation. Guest transition moments aligned parallel to the cyclodextrin cavity axis exhibit a positive induced circular dichroism, while a negative dichroism is observed for those moments perpendicular to the cavity axis.^21,31^ Induced circular dichroism of O-II in the presence of γ-CD shows a positive dichroism for the 477 nm band and a negative dichroism for the 525 nm band.^10,13,32^ It should be noted that there is a discrepancy of about 5–10 nm between the observed bands^10,13,32^ to those calculated by Reeves et al.^20^

**Figure 7:**
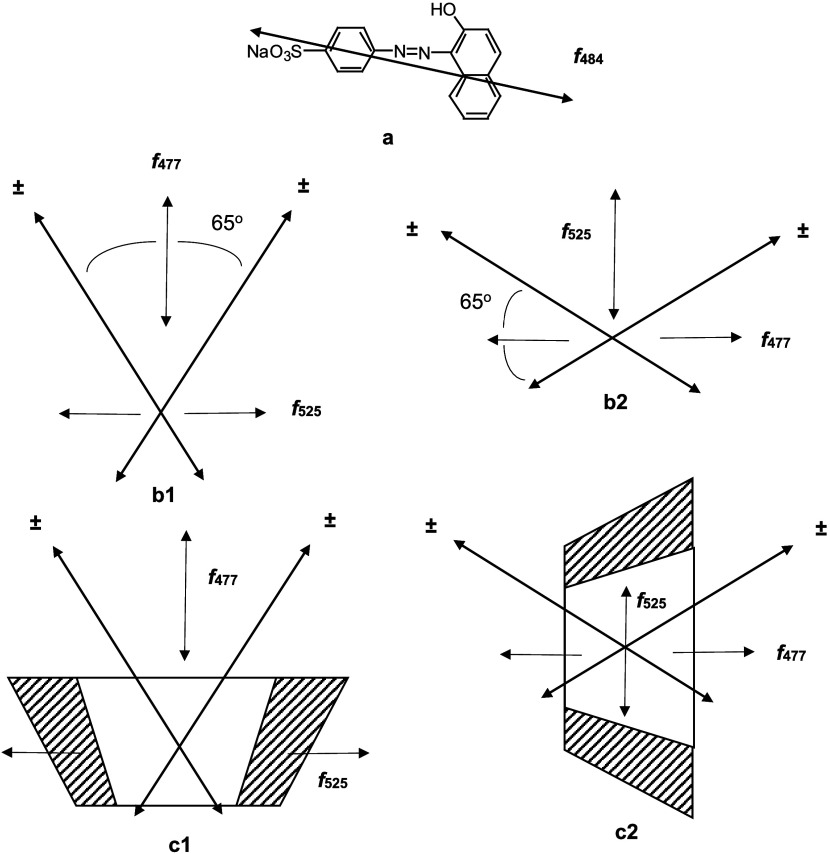
(a) Transition dipole moment orientation for O-II.^32^ (b) Proposed O-II dimer geometries and their respective orientations (c) within the γ-CD cavity, where ± represents the charged portion of O-II.

The transition dipole moment for the 484 nm band for O-II has been calculated^32^ to be oriented along the long axis of the molecule (shown in Figure 7a), which is supported by linear dichroism measurements of stretched poly(vinyl alcohol) films dyed with O-II.^13^ Based on the transition moment orientation for the O-II monomer and the 65° angle between monomer moments for the O-II dimer, two possible O-II dimer geometries are shown in Figure 7b, which attempt to qualitatively balance the π–π overlap and Coulombic repulsions of the NaSO_3_ moieties. While considering the orientation of the 477 and 525 nm moments and their respective signs found from induced circular dichroism, the orientation of the O-II dimers within the γ-CD cavity is shown in Figure 7c. It should be noted that the O-II/γ-CD complexes in Figure 7c are only schematics and are not based on any molecular modeling calculations. These geometries are at this time only considered as candidates, for subsequent projection of the self-assembly mechanism for the anisotropic phase of the lyotropic liquid crystalline phase of the O-II/γ-CD system. Additionally, both geometries should be considered to likely have the O-II naphthol moiety residing within the CD cavity according to NMR^33^ and femtochemistry studies.^34^ Owing to the amphiphilic nature of O-II, the structures shown in Figure 7c1 may occur in a similar manner as to how charged surfactants orient during micelle formation. However, it should be noted that Clarke et al.^13^ suggested that the O-II dimer might be an oblique, yet antiparallel (to relieve Coulombic repulsions), dimer within the cavity, whose description is similar to that drawn schematically in Figure 7c2. Elucidating the more likely arrangement could be the subject of future molecular modeling studies.

### Proposed Superstructure of O-II/γ-CD

**Figure 8:**
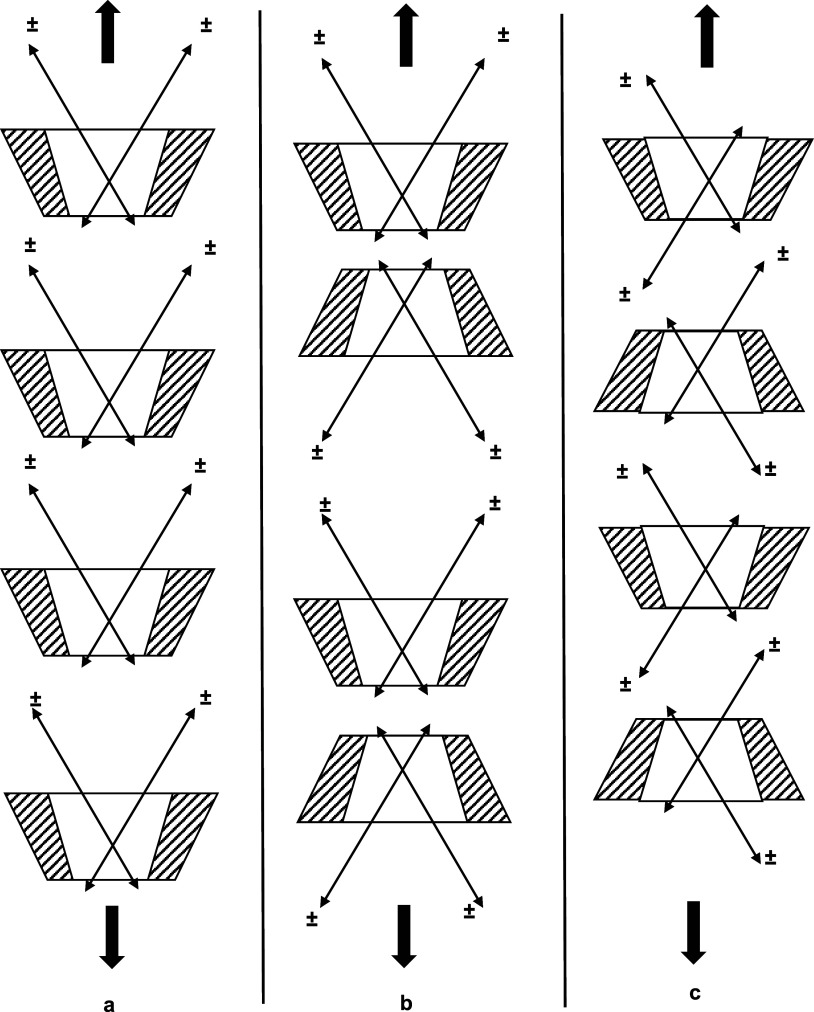
Proposed rodlike superstructure assemblies based on Figure 7c1 (a, b) and Figure 7c2 (c) that may extend further in the directions marked with an arrow.

Based on the observations discussed earlier, it is evident that the fundamental “building block” for the system leading to a lyotropic liquid crystalline phase is two O-II molecules in a γ-CD cavity. We have proposed two geometries that are consistent with the experimental observations. As already noted, in order for the system to exhibit a liquid crystalline phase, these “building blocks” must assemble into a rodlike structure, which above a critical concentration can lead to a liquid crystalline phase^11^ in solution. Figure 8 illustrates three possible superstructures based on the 2:1 O-II/γ-CD complex, providing a rodlike superstructure. This is consistent with the nanotube structures which are believed to form in solution when certain cyclodextrins complex with either 2,5-diphenyloxazole^35,36^ or 1,6-diphenyl-1,3,5-hexatriene.^37−39^

**Figure 9:**
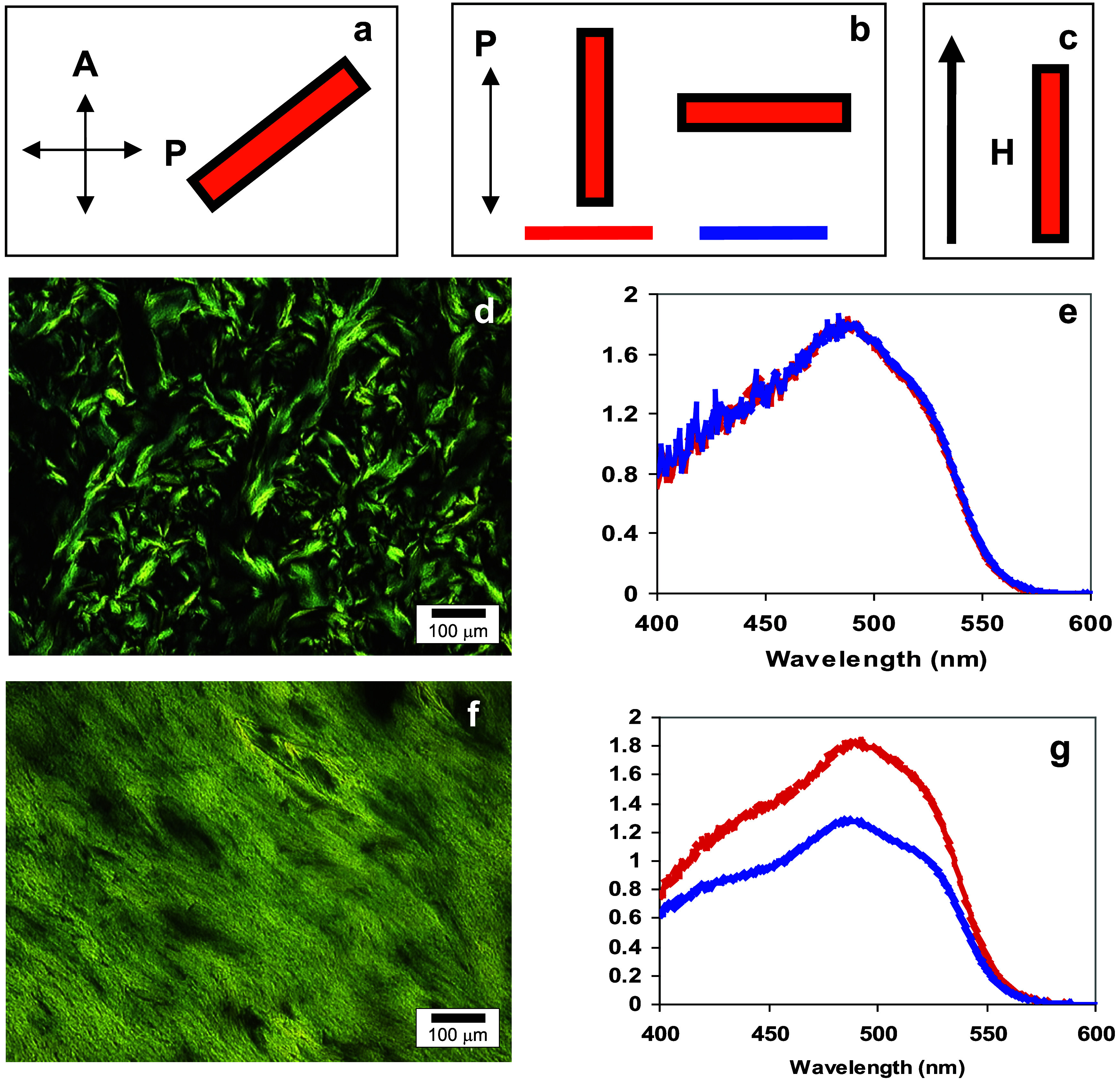
Sample: Anisotropic phase from 0.05 M Orange II with 0.05 M γ-cyclodextrin. (a) Sample cell orientation with respect to polarizers for optical micrographs. (b) Sample cell orientation color-coded with respect to the polarized incident light for linear dichroism spectra. (c) Sample cell orientation with respect to the magnetic field. (d) Optical micrograph and (e) linear dichroism spectra for the sample before exposure to the magnetic field. (f) Optical micrograph and (g) linear dichroism spectra for the sample after exposure to the magnetic field.

Liquid crystals align in the direction of an applied magnetic field if the diamagnetic susceptibility is positive.^11^ In an effort to confirm that the superstructures are rodlike, an aqueous solution of 0.05 M O-II/0.05 M γ-CD (in the fully liquid crystalline phase) between two glass plates separated by a 40 μm spacer was subjected to a magnetic field (9.4 T) for 16 h, with the sample plane parallel to the magnetic field direction. The linear dichroism and polarized light microscopy observations indicated that the presumed rodlike structure had indeed aligned along the applied magnetic field direction (Figure 9).

## Conclusions

Fluorescence spectroscopy and conductivity titrations for the O-II/γ-CD system provide further support for the inclusion of an O-II dimer within the γ-CD cavity. Candidate dimer orientations within the γ-CD cavity are provided based on molecular exciton theory and induced circular dichroism data. Of the two candidates shown, it may be possible that an oblique parallel arrangement of O-II monomers assemblies into a dimer that is included in the γ-CD cavity, as an alternate option to that suspected by Clarke et al.^13^ However, both of these geometries should be further investigated with molecular modeling studies. Finally, we propose a superstructure that forms a rodlike structure based on the 2:1 Complex between O-II and γ-cyclodextrin.
